# Selections and Abstracts

**Published:** 1906-07

**Authors:** 


### SELECTIONS AND ABSTRACTS.

### Prayer of a Physician, Prominent in Alexandria in Egypt in the Twelfth Century.

The following interesting prayer was read by Prof. Leonard Weber at the annual dinner of the faculty of the New York Post-Graduate Medical School and Hospital.

"O Lord, thou hast most wisely fashioned man's body, thousands upon thousands of organs hast thou joined in it to work incessantly in order to build up and preserve the beautiful whole, the dwelling of the immortal. In perfect order and union they will perform their functions, but when their harmonious action is interrupted by the fragility of the constructive material or the perversity of the passions, the forces will antagonize each other and the body may perish. And thou sendest the warning messengers, diseases, to man, to show the threatening danger and stir him to try and avert it.

"Thy earth, thy rivers, thy mountains are full of healing substances which have the power to mitigate suffering and to prevent the destruction of thy creatures. And to man thou hast given wisdom to study the body and understand the organs in their order as well as disorder; also to seek and find those remedies and prove and prepare them according to the indications for disease.

"By thy grace I have been called to watch over sickness and health of men, and I make ready now to start in the professional work of the day. Be my strength, good Lord, in this great undertaking, and bless my work that it may be good.

"Let me be filled with love of man and my art, and do not let desire for gain or position or fame interfere with my duties.

"Preserve the strength of body and mind that I may always and equally serve the poor and the rich, the good and the bad, the friend and the enemy, for all of them are thy creatures.

"Let my reason be steady and sound, that I may well observe what is before me and truly surmise what is hidden. Let my mind not be confused and overlook what is present, or go beyond that which can be actually seen and proved into the territory of the speculative, the invisible. For, fine and scarcely traceable are the border-lines of the great art in caring for the health and life of man.

"Let me be always myself and my attention fully concentrated, at the bedside nothing foreign must disturb it, so that all the experience and insight I may have will be at my command in the case before me.

"Fill my patients with confidence in myself and my art, and with obedience to my advice.

"Keep away, O Lord, from the sick chamber every quack and the whole army of advisory relatives and the over-wise nurse; they constitute a cruel set of people, who, in their vanity, may spoil the best work of medical art, and not infrequently assist the disease in destroying the patient.

"When wiser counsellors than myself are ready to correct and improve my knowledge, let me gratefully receive their counsel, for art is broad and wide, and no one man can know all.

"But should the vain and unwise find fault with my skill, then make me firm as steel to stick to the truth and right of my position, and maintain the same against the greater fame and age of others.

"Give me patience and humility in dealing with such of my professional brethren as, being proud of their long years of practice, may try to push me aside and belittle my knowledge. I will profit from the wisdom which they may have to offer, but will also try and bear with their haughtiness; for they are old in years, and old age does not always control the passions—and I also hope to grow old in walking before thee, O Lord.

"Whatever I have or may possess will suffice me, but not as to science and art. Let never the thought arise in me: 'I have of knowledge enough,' but extend to me the power, the leisure, the burning desire to correct and adjust my accomplishments and add to them steadily. Great is our art, but the power and extent of the human mind is also vast and its limitation unknowable. Further and further we advance, but in the science of yesterday we find errors to-day, and that of to-day will be questioned to-morrow.

"Give me thy gracious help and protection, O Lord, in the work to which I will go now, that it may prove a blessing to those who will be entrusted to my care."

NOTE.—This beautiful and noble composition appeared first in the *German Museum Magazine,* 1785; a reprint of the same was published in the *Deutsche Med. Woch.* some years ago, and has been translated for the *Post-Graduate Medical Journal* by Leonard Weber, M.D.—*The Post-Graduate, May,* 1906.

---

### DR. JOHN C. LEGRANDE.

As we are ready to go to press we are saddened by the startling intelligence that our friend and colleague, Dr. John C. LeGrande, editor of the *Alabama Medical Journal,* is dead. While his health had not been good for some time, no one suspected that the end was so near.

Dr. LeGrande was widely known among the profession in Alabama by virtue of his editorial employment and his influential connection with the Medical Association of the State. While not a great and erudite editor, on account of his conscientious devotion to advancement along professional lines, he exerted a wide and wholesome influence; and every doctor who knew him will regret his death. Devoted to the State Medical Association, he was keenly alive to every proposition for professional progress. Personally he was a genial gentleman, and no one had a higher regard for him than the writer.

John Clark LeGrande, M.D., was born at White Plains, Calhoun county, Alabama, December 6, 1854, and was the son of J. C. and Martha A. (Watson) LeGrande. He spent the first eighteen years of his life on his father's plantation and in attendance on the common schools. He subsequently attended a high school in Georgia, read medicine and graduated from the Atlanta

Medical College in the spring of 1880. He began the practice of his profession in his native county, and was located at Weaver's for three years. In the fall of 1883 he moved to Anniston, and speedily took rank among the foremost of his profession. He was one of the charter members of the Calhoun County Medical Society, organized April 30, 1880, and was its secretary for nine years. He was president of this society for two terms. He was also assistant health officer for Calhoun county.

He removed in 1897 to Birmingham, where he built up a good practice. He was married December 2, 1880, to Janie Lee Ayers, of Carnesville, Ga. They have had five children, named respectively, Mary Ruth, Bessie, Annie F., Boyd G. and Howard H. Dr. LeGrande was a member of the Methodist Episcopal Church, South, and also belonged to several secret orders.

The father of Dr. LeGrande removed from Georgia to Alabama and settled near White Plains in 1852. He was engaged in teaching for several years; entered the Confederate army in 1862, and died in Atlanta in April, 1864. He reared a family of four sons and two daughters. Dr. LeGrande's grandfather was a native of South Carolina.

The LeGrandes were French Huguenots. Since moving to Birmingham Dr. LeGrande's wife died, in 1899. In 1896 Dr. LeGrande was vice-president of the American Medical Association, being elected in Baltimore in 1895. He presided in Atlanta over the meeting of the association, during the greater part of the session held in June, 1896.

In April, 1899, in Mobile, he was elected president of the State Medical Association of Alabama, and presided at the meeting held in Montgomery in 1900. In 1901 he was elected president of the Tri-State Medical Society of Tennessee, Georgia and Alabama, at Nashville, Tenn., and presided at the session held in Birmingham in 1902. Dr. LeGrande was editor and proprietor of the *Alabama Medical Journal,* which he conducted successfully for fifteen years. He was secretary and treasurer of the Birmingham Medical College, and had full charge of all the business affairs of that institution. He was also professor of obstetrics in the college.

Those who regularly attend the annual sessions of the Medical Association of the State of Alabama will miss Dr. LeGrande. His dignified yet genial greeting, and his participation in the discussions before that body made him a familiar figure. We sadly regret his demise, and tender to his loved ones our sincere sympathy.—*Editorial, Mobile Med. & Surg. Journal, March, 1906.*

---

### HISTORY OF THE GOLD-HEADED CANE.

In opening the symposium upon the Gold-Headed Cane before the Johns Hopkins Historical Club, January 29, 1906, Dr. McCrae gave its history as follows:

Among the many treasures of the College of Physicians in London not the least interesting is that known as the Gold-Headed Cane. This made its appearance in medical circles probably about the year 1689 and for one hundred and thirty-six years was carried by a leading London practitioner, during this time passing through the hands of Radcliffe, Mead, Askew, the two Pitcairns, and Baillie. The shape of the handle of the cane is rather unusual for the time when it was made, as at that period the head of the physician's cane was generally round and contained a cavity in which aromatic substances were carried, the inhaling of which was thought to prevent contagion. The first owner of the cane, Radcliffe, was a man always impatient of convention, and it is possible that he adopted the unusual shape to be different from the rest of the profession. The cane came into possession of the College as a gift from Mrs. Baillie, the widow of the last of the series who carried it.

The book which is known as "The Gold-Headed Cane" is written in the form of what may be termed an autobiography. The cane is represented as giving an account of the lives of the men who carried it and the more interesting events in which they were concerned. The book was published without the author's name, but was written by Dr. William Macmichael (1784-1839). The first edition was published in 1827 and the second in 1828. The third edition, which was published in 1884, was edited by William Munk, and in it many additions were made. The book is written in a chatty, conversational style, deals with many points in the lives of its possessors and also refers to many of the happenings of the times.

The first possessor of the cane, John Radcliffe (1650-1714), is discussed elsewhere. From him the cane passed to Richard Mead (1673-1754), one of the most interesting figures in British medicine. He studied classics at Utrecht, physic at Leyden, where he was a student with Boerhaave, with whom he kept up an intimate friendship, and graduated at Padua in 1695. He settled in London and was shortly afterwards appointed to the staff of St. Thomas' Hospital. He was a great collector of books, prints, pictures, coins, and gems, as well as Oriental, Greek, and Latin manuscripts. In the chapter of the Gold-Headed Cane dealing with his life we are brought into contact with many of the interesting people of the time—Sir Hans Sloane, who founded the British Museum; Dr. George Cheyne, who pleaded for a simpler life even at that day; Lady Mary Wortley Montague, and the introduction of the inoculation for smallpox. Accounts are given of meetings at Mead's house in Great Ormond street, where the Children's Hospital now stands, in which Arbuthnot and Pope figure, and also a description of the last illness of Sir Isaac Newton. Medically, Mead was especially interested in snake venoms and had also much to do with the introduction of inoculation for smallpox. It is interesting to remember that he probably had considerable influence on the founding of Guy's Hospital, as he and Guy were great friends and Mead was consulted as to the best way in which Guy could use his fortune for the establishment of a medical charity. Mead was a great patron of literature and art and has been termed the Mæcenas of his day. It is rather a commentary on what makes for fame that Radcliffe, who did comparatively little during his life, is so well remembered through his bequests, while Mead, who did so much during his life, left little behind him by which he is remembered.

From Mead the cane passed to Anthony Askew (1722-1774), a graduate of Cambridge, who studied at Leyden and subsequently spent many years in traveling abroad. He settled in London, was attached to St. Bartholomew's Hospital and became a very intimate friend of Mead. He is perhaps better known as a classical scholar than as a physician. He was a great book collector, and it was even said that it was difficult to get into his house on account of the halls being filled with books. Dr. Cushing will speak more especially of his library. After the death of Askew the cane passed into the possession of Dr. William Pitcairn (1711-1791). He belonged to the same family as Archibald Pitcairn, of Edinburgh, who at one time occupied the chair of physic in Leyden, and was a great exponent of the mechanical school of medicine. William Pitcairn studied at Leyden and took his degree at Rheims. He was associated with St. Bartholomew's Hospital and was president of the College of Physicians for many years. During the latter part of his life he gave up active practice and transferred the cane to his nephew, Dr. David Pitcairn (1749-1809). He was also attached to St. Bartholomew's and is said to have been the first to recognize the relationship between acute rheumatic fever and endocarditis. He died of edema of the glottis, and the description of his condition is said to be one of the earliest in which the account of an autopsy was given.

From Pitcairn the cane passed into the hands of Matthew Baillie (1761-1823). About this time the cane ceased to be considered a necessary appendage of the physician, and after the death of Baillie in 1823 the cane was presented by his widow to the college, where it was deposited in 1825. At this point the history of the cane as given in the first and second editions of the work comes to an end, but in the third edition the cane is represented as continuing the history of the college and of some of its most prominent members after 1825. There is also a note regarding many of the portraits and busts in the college.

In this hasty review reference has been made to only a few of the many interesting points in the history of the Gold-Headed Cane. It will probably not be possible for all of you to pick up copies, but those of you who read it will find many facts regarding some of our predecessors which will stimulate interest in the history of the profession, one of the objects of this club.—*Johns Hopkins Hosp. Bul., May, 1906.*

---

### THE TREATMENT OF DYSPEPSIA.

In the *British Medical Journal* of November 25, 1905, Hutchison writes upon this subject.

In dealing with this part of the subject he departs from the plan he has hitherto adopted, and instead of considering the ways by which one can correct the disorder in each function separately, he takes up the agents which are at our disposal for these purposes, and indicates very briefly the manner in which they can be used to influence favorably any disorder or group of disorders of function which the stomach may exhibit. He gives only a mere sketch of rational gastric therapeutics.

The agents of which we can avail ourselves in any attempt to regulate the functions of the stomach are these:

1. General measures—for example, rest, exercise, massage, hydrotherapy, electricity.
2. Dietetic means.
3. Drugs.
4. Operation.

We may now consider very briefly the special limitations and uses of each of these agents.

1. *General Measures.*—Our study of the causes which produce disturbance of function in the stomach has shown us what a very large part nervous influences play in the process. It follows from this that we must in most cases look first to agents which act upon the nervous system when we are considering the treatment of a case of dyspepsia. Of such agents none is more potent than rest, physical and mental. By rest is not meant rest of the stomach itself, such as one obtains by exclusive rectal feeding—for that is but rarely required in the treatment of functional dyspepsia—but general rest of the body as a whole, rest in bed. So potent is this agent that it suffices in many cases to send a dyspeptic patient to bed to relieve him at once of most of his complaints. Yet, in the opinion of the author, we do not make nearly as much use as we might of this simple means; we do not send our dyspeptic patients to bed half often enough. Equally important is mental rest and the liberation of an overtaxed brain from the cares and worries of an active business or profession life. Many of our readers have experienced for themselves the extraordinary improvement in all the functions of the stomach which is brought about by going away for a holiday and casting off for a time the preoccupations and anxieties of practice. Exercise is a therapeutic agent of far less utility than rest, and it is much more difficult to know when to prescribe it. The author believes it to be especially indicated in some cases of depression of the motor and secretory functions, just as rest is chiefly to be used in cases of exaggerated sensibility.

Of all forms of exercise, riding is probably the best—was it not Palmerston who said that "There is nothing like the outside of a horse for the inside of a man?"—but walking, especially in a mountainous country, is very serviceable (it was by means of tours in the Alps that Huxley, for instance, kept his habitual dyspepsia at bay), and golf, which combines with walking some exercise of the abdominal muscles, is also very helpful. Something, also, is said for the use of the voice. Loud reading—clara lectio—was a favorite prescription of the Roman physicians, and its mode of action probably consists in compression or massage of the stomach by the diaphragm favoring motility. Certainly any one who has ever done much lecturing knows what an appetizer it is.

Massage—by which is meant especially abdominal massage—is a great aid in promoting one function of the stomach, namely, motility, but it is doubtful if it has any influence on secretion; in hyperesthesia it is positively harmful. Hence its use must chiefly be confined to atonic cases. With hydrotherapy as a means of treatment the author deals briefly, but he speaks confidently of the value of hot fomentations in allaying hyperesthesia, and fancies that suitable douching might greatly stimulate motility.

About the value of electricity as a therapeutic aid the author speaks hesitatingly, for it is so difficult to get the treatment properly applied by honest and competent persons and at a cost which most patients can afford. He is prepared to believe, however, that it might aid greatly in stimulating the movements of the stomach, and sees no reason why it should not be of as much use in diminishing hyperesthesia as it sometimes is in painful affections elsewhere, such as sciatica. The difficulty about most of those so-called physical methods of treatment is that of getting them carried out efficiently and persistently. We are greatly in need of institutions where such treatment could be obtained under skilled supervision and at reasonable cost.

2. *Dietetic Means.*—In the opinion of the author errors of diet play but a small part in the production of dyspepsia, and he adds that dietetic means are not really of much value in its cure. We can do much to palliate a patient's symptoms by adjusting the diet so as to burden the affected function as little as possible, but this is not to cure him in the proper sense. Meanwhile, the stomach may recover spontaneously, or the error of function be cured by other menas, but a dyspeptic has not really recovered until he can digest food like other people. So long as he requires to observe rules of diet, even although he is perfectly free from symptoms, so long is he a sick man.

Without discussing the principles of dietetic treatment, the author states in general terms that where motility is chiefly affected the mechanical form of the food is of more importance than its chemical composition, but that where secretion is perturbed the reverse is the case, whilst in hyperesthesia the form and composition and temperature of the food have all to be considered. Good cooking is of importance to the dyspeptic in so far as it affects the form and consistency of the food, and attractive serving of meals is of the greatest help in promoting secretion.

Amongst dietetic means must be reckoned alcohol, which in moderate doses is a powerful stimulant both of the secretory and motor functions. Its use should therefore be reserved for the atonic cases.

3. *Drugs.*—The author quotes an eminent physician, now deceased, who used to sum up his advice to his students regarding the use of drugs in dyspepsia in these simple words: "Gentlemen, if you fail with your alkalies try your acids." And is that not pretty much what most of us do? If we fail with our bismuth we try our rhubarb, and if we fail with rhubarb we try pepsin, and so on—groping in the dark, in the hope that we may find something that will do good. From such treatment no mental satisfaction can be obtained, but by adopting a classification of cases our choice and use of drugs at once become easy and rational. One has merely to determine what function is perverted, and in what direction, and then to make use of the drugs which are known to act upon that function. Into details of the administration of drugs in dyspepsia the author does not enter, simply suggesting a list of those which are of most help in each form of disturbed function, as follows:

*Increased Secretion.*—Belladonna undoubtedly lessens gastric secretion; but as its administration is attended by inconveniences, it is usually better to content oneself with neutralizing the excess of secretion by earthy carbonates, such as carbonate of magnesia; astringents, such as tannic acid, also lessen secretion, and the stomach may be washed out with solutions of them in some cases.

*Diminished Secretion.*—Secretion may be promoted by the use of soluble alkalies—for example, bicarbonate of soda—and bitters before meals; also by stimulating drugs, such as capsicum and ginger. It may be supplemented by hydrochloric acid given an hour after food. Pepsin preparations are rarely needed, and are of doubtful value.

*Diminished Motility.*—The movements of the stomach may be stimulated by ipecacuanha, strychnine, quinine, and probably also by hydrochloric acid.

Increased motility is best met by neutralizing the excess of acid which causes it (*vide supra*). Also by the sedative drugs useful in hyperesthesia.

Increased sensibility is allayed by bismuth, hyoscyamus, bromides, chloral, cocaine, hydrocyanic acid, opium, and chloroform, and by agents which neutralize hyperacidity. In the idiopathic (gastralgic) form it may be treated by arsenic, the coal-tar derivatives, by nerve tonics, such as quinine and strychnine, and by bromides.—*Therapeutic Gazette, April,* 1906.

---

THE ADMINISTRATION OF MEDICINE DURING THE NIGHT.

René Laufer (*Bulletin général de thérapeutique,* January 23, 1906) calls attention to the fact that not only are the bodily functions modified at night, but diseases are affected in their course. Thus, some are manifested most frequently at night, like asthma; or may appear both by day and night, but are worse at night, like epilepsy, or painful syphilitic manifestations. Others continue day and night, but are modified and perhaps more acute at night, like articular rheumatism. This pathology of night may be rationally opposed by a nocturnal therapeutics. In rheumatism, for instance, the evolution of the disease progresses steadily during the night as well as the day, and the remedies should likewise be continued. In a case of asthma, which regularly occurred between midnight and three o'clock in the morning, the administiation of one gramme of potassium iodide on going to bed prevented the recurrence of the attacks. In a case of nocturnal epilepsy, the bromides were given three times during the day, and also at 11 o'clock at night and about 1 o'clock in the morning (in a little milk). Although previously the patient had had seven or eight attacks a week (two or three during the day, the rest at night), he had during the first week of this treatment no attack in the day and only one at night. In the fourth week there was only one seizure, and after that for four weeks there were no attacks at all. Besides the potassium bromide in doses of 0.50 gramme, three times a day and twice at night, he was given a diet free from sodium chloride, and for the time was kept on a strict milk diet. In cases of acute rheumatism, where the sodium salicylate treatment (3 grammes during the day, in six doses at intervals of two hours, and 2 grammes at night, in three doses, at midnight, 3 and 6 o'clock) was given, and they were kept on a milk diet, the cure was obtained in much less time than by the ordinary method. The same principle of treatment is applicable to syphilitic neuralgias, to whooping-cough, bronchitis, enteritis, etc. Laufer believes that remedies are more effective at night because absorption is probably more rapid, and elimination on the contrary is less active. Owing to certain conditions, also, it is probable that the toxicity of remedies is greater at night when the stomach and intestines are comparatively empty. The use of an alarm clock enables patients to take the medicines themselves, but some awake spontaneously. The administration of remedies during the night in acute rheumatism was advocated by Huchard, in his *Practice,* a number of years ago, and is now generally followed.—*N. Y. and Phil. Medical Journal, March 3.*

---

### A NEW TECHNIQUE FOR BREAST AMPUTATION.

Believing that his method has certain elements of advantage, as well as originality, Jackson (*Journal A. M. A.,* March 3), with many excellent photographs and cuts, describes his operation for removal of the breast, and for it claims these advantages:

First: The flap forms a covering for the chest defect, as a rule without any tension, and thus almost entirely obviates the necessity of grafting, which is so frequent in other methods. In fact, I have not found any patient operated on by my method who required grafting. Of course, this operation is not intended for cases in which there has been extensive previous ulceration or in which there is not healthy tissue for a flap of any character.

Second: The drawing of the skin up to the arm does away with the axillary fossa, and thus with the large space which Nature would have to obliterate by formation of scar tissue, with the resultant pressure on the axillary vessels and nerves.

These first points are the ones which would probably be most striking to one who has not seen the operation.

Third: The ligation of all vessels at their nearest point of origin does away with the use of a large number of hemostatic forceps, which cause loss of time, to say nothing of the inconvenience of having a large number of instruments in one's way. I have in no instance used more than one dozen forceps in this operation, and can usually do the work with about six. The operation is thus shortened, so that, as a rule, I find that it requires from forty minutes to one hour or thereabouts to complete it. In fact, I have never run beyond an hour, even doing the operation slowly, as I have in most cases, for the purpose of demonstrating this new technic, and I have done the operation in forty minutes.

Fourth: The most noticeable feature to the onlooker when the operation is done in the manner described probably is the marked absence of hemorrhage, so that it can almost be called a bloodless operation.

Fifth: The entire technical portion of the operation is completed before the chest is exposed by removal of the breast; therefore long exposure of an enormous area of raw chest surface, with the attendant shock, is done away with. As soon, in fact, as the breast is removed the wound is ready to be closed.

---

Reprint from the *American Journal of Surgery*, August, 1905.

### MODERN CIVILIZATION AS A FACTOR IN CAUSING DISEASES OF WOMEN.

BY N. H. KASSABIAN, A.B., M.D., Coopersville, Mich.

A woman physically perfect is certainly a unique creature in modern times. Our civilization has contributed very extensively to the causation of female maladies which we are so frequently called upon to treat. Our modes of living and dressing exert a very deleterious influence upon the normal functions of the pelvic organs, not to mention the hepatic compressions and subsequent displacements resulting as a natural consequence of tight lacing practiced by the most humble devotees of the temple of fashion. We are a very progressive race, taking herculean steps towards a higher civilization; but it is a deplorable fact that the more we learn the more we ignore the most fundamental laws of hygiene. The immediate effects of tight-lacing are, that abdominal and spinal muscles are seldom brought into play, so they become atrophied. The viscera are thus compressed and displaced, and the full play of the abdominal wall and the descent of the diaphragm are interfered with and the venous blood is hindered in its return to the heart. This obstruction of the circulation and the constipation from which women habitually suffer lead to a permanent dilatation of the pelvic veins, a very fruitful source of diseases of the genital organs. And again, customs demand the protection by veil and gloves from the rays of the sun, and the woman soon becomes as bleached as a well-cultivated celery stalk. As the blood needs the direct chemical effect of the sun-light on the skin an anemia is established. This state of the blood is a potent factor in the generation of all the diseases depending on impaired nutrition and entails conditions likely to baffle all medical efforts at their removal during the menstrual life of the woman.

Before the altar of fashion and so-called society our girls, while entering to puberty, have to comply with the dictates of the times and at the period of life when the young girl's whole nerve force is taxed for the full development of her organs of generation this force is deflected by hard study, and it may be for the acquirement of some accomplishment which, in all probability, will be forgotten or laid aside after marriage. She is subjected to the emotional influence of music and light literature, which are capable of arresting the normal development of the uterus and its accessory organs.

During my travels in the Orient I have been very much surprised to see, specially among races who do not comply with the trivial demands of modern civilization as their sisters do in Occidental countries, young ladies grown to womanhood and into maturity experiencing no menstrual irregularities whatever, giving birth to children with normal labor, and through all their maternal life they do not seem to have any especial need to consult a physician. I do not dare, still I take the liberty to state that modern civilization and society have immensely contributed to the creation of gynecology, and the majority of patients who make a daily pilgrimage to a gynecologist's office are the poor and pitiable victims of a misdirected and misinterpreted civilization.

There are very few maladies which we are called upon to prescribe for as much as for menstrual disorders. A thorough examination with a view of ascertaining, if possible, any malposition of the genital organs should be insisted upon. The least abnormality should have proper attention and local as well as constitutional treatment. Anemia and chlorosis should have their attention, as they almost invariably play a conspicuous part in causing menstrual irregularities. In the form of Ergoapiol (Smith), I believe we possess a remedial agent that combines the most effective ingredients with which we can combat the majority of menstrual irregularities.

Clinical observations with this preparation are as follows:

CASE No. I.—Miss A. B. Age sixteen. Parents both living and in excellent health. She has not had any serious sickness, although on account of there being a tubercular diathesis in the family the least abnormality in the functions of any of the organs is looked upon with suspicion and at once the advice of the family physician is sought. So when Mrs. A. B. noticed that her daughter had an irregular menstruation accompanied with dysmenorrhea she consulted me in her daughter's behalf. I prescribed for her Ergoapiol (Smith) capsules, directing her to give one three to four times a day with milk for three or four days before the expected menstrual period. They complied with my directions very faithfully, and after a few weeks they reported that her menstrual disorders had been satisfactorily regulated.

CASE No. II.—Mrs. B. Age thirty-eight. Has two children. Mother died from the result of an operation for appendicitis. Father living and in good health. Patient has been anemic for many years, her hemoglobin test showing only 40 per cent. Has at present prolapse uteri to a slight degree, some perineal laceration and endometritis. Digestion is at fault part of the time, bowels constipated, ringing and buzzing in the head. Menstruates every three weeks. During the first day of the period it is very painful and she flows very profusely, its duration being from a week to ten days. I prescribed for her Ergoapiol (Smith) capsules, one three to four times a day, a day or two before the expected menstrual period, to be continued until menses appears, then to discontinue the capsules for three days, and recontinue, commencing on the third or fourth day. The therapeutic action of the remedy was all that could be expected. The dysmenorrhea, which was always a conspicuous feature, seemed to yield after the administration of the first few capsules. She menstruated quite easily and the duration was very moderate.

CASE No. III.—Mrs. ——. Age thirty-eight. Has two children, youngest ten years of age. Lacerated perineum and cervix with its sequelæ, *i. e.*, endometritis, leucorrhea, bearing-down pains in front and in the back. Uterus extraordinarily heavy and patulous. Lack of tonicity in the uterine muscles and ligaments. I curetted her, expecting some benefit from the procedure. Uterus was packed with iodoform gauze, which was removed next day; but a hemorrhage still persisted for a few days, as it seemed to be due to a deficiency of contractility of the uterine muscles. I prescribed 2 grs. of sulp. quinine in connection with Ergoapiol (Smith) capsules, one three to four times a day, preferably taken with some milk. The remedies had the desired therapeutic action, checking the uterine hemorrhage by increasing the tone of the muscles and at the same time controlling the blood vessels supplying the endometrium.

CASE No. IV.—Mrs. ——. Age thirty-nine, Two children, youngest eight years old. A sufferer from digestive disorders, anemia, and of late, while approaching menopause, she has been having considerable difficulty with her menstruations. At times very profuse and exceedingly painful, and again very scant and period prolonged. Having tried a number of preparations without any appreciable benefit to her, decided to use Ergoapiol (Smith). She was directed to use a capsule three to four times a day, commencing two or three days before the expected menstrual period. After using this preparation for a little while she reported that she "had not suffered any pains or inconvenience since she began taking those capsules."

CASE No. V.—Mrs. ——. Age thirty-nine. Two children, youngest twelve years of age. An exceedingly anemic patient, hemoglobin tests showing 55 per cent. Had a miscarriage recently, and after the uterus was free from any of the products of gestation she continued having a profuse hemorrhage in spite of the intra-uterine douches and packing I used every day. Finally, having concluded that this hemorrhage was due to atonic condition of the uterus, I ordered for her Ergoapiol (Smith), one capsule, to be taken from three to four times a day. Her hemorrhage decidedly ceased in course of forty-eight hours, and she made a very gratifying recovery.

CASE No. VI.—Mrs. ——. Age thirty-eight. Married eighteen years, four children, youngest four years old. During her menstruation she flows so profusely that she is frightened of a fatal termination. I prescribed for her Ergoapiol (Smith) capsules, instructing her to take one after appearances of menstruation, three to four times a day for two or three days until the menstrual flow was controlled. During her subsequent visits to my office I was very much pleased to learn that she has had no trouble to speak of with her menstrual periods while taking the capsules that I prescribed for her.

### THE CHICAGO STOCKYARDS.

The question of pure food is a vital one, and only this year did Congress see fit to pass a law making for the betterment of food sold to the public. Recently a novel has appeared which claims to show the lax methods in vogue in the Chicago stockyards. These yards were some time ago severely condemned by a commissioner sent to this country by the London *Lancet.*. It is claimed that the government inspectors allow all sorts of diseased cattle to pass into the public supply. It is bad enough that the public should be at the mercy of the so-called beef trust in the way of high prices, but when they have tainted meat given them it is high time that strenuous efforts be made to insure a proper standard of meat inspection.—*Medical Age, May 25, 1906.*

---

IN the performance of high tracheotomy a great deal of room can be gained by dividing transversely the fascia that extends upward from the thyroid.—*American Journal of Surgery.*

---

The report of the dispensary physician, University of Maryland Hospital, shows 28,028 cases treated during the year ended April 1, namely, 7,163 new and 20,865 old cases.

